# An omics-based characterization of *Wolfiporia cocos* reveals three *CYP450* members involved in the biosynthetic pathway of pachymic acid

**DOI:** 10.1038/s42003-024-06323-1

**Published:** 2024-05-30

**Authors:** Heping Liu, Naliang Jing, Fengfeng Li, Keyue Wang, Jing Tang, Qin Zhao, Yipeng Zhang, Hamza Armghan Noushahi, Ran Xu, Xuekui Wang, Wenjun Zhu, Shengqiu Feng, Shaohua Shu, Zhinan Mei

**Affiliations:** 1 College of Plant Science and Technology, Huazhong Agricultural University, Wuhan, Hubei Province China; 2 School of Life Science and Technology, Wuhan Polytechnic University, Wuhan, Hubei Province China

**Keywords:** Enzymes, Fungal genomics, Fungal biology

## Abstract

*Wolfiporia cocos* is a medicinal mushroom used in China. It biosynthesizes pachymic acid (PA), a main therapeutic triterpene associated with therapies. Nowadays, the unknown PA biosynthesis leads to difficulties in increasing its content in *W. cocos*. Herein, we report sequencing, assembling, and characterization of the genome and several transcriptomes of *W. cocos*. Sequence mining determined candidate genes that encode lanosterol synthase, sterol O-acyltransferase, and sterol C-24 methyltransferase likely involved in the steps from lanosterol to PA. Gene cluster analysis identified four CYP450 cDNAs likely involved in the biosynthesis of PA, namely *WcCYP64-1*, *WcCYP64-2*, *WcCYP52*, and *WcCYP_FUM15*, which were subjected to both overexpression and silencing in mycelia. The overexpression of each of *WcCYP64-1*, *WcCYP52* and *WcCYP_FUM15* increased the content of PA, 16α-hydroxytrametenolic acid, eburicoic acid, and tumulosic acid, while the silencing of each gene either significantly or slightly decreased the contents of these four compounds, indicating their involvement in the PA biosynthesis. In addition, different temperatures affected the expression of these genes and the formation of PA. By contrast, the overexpression and silencing of *WcCYP64-2* did not alter the formation of these compounds. Taken together, these findings determine more potential steps in the biosynthetic pathway of PA for metabolic engineering.

## Abstract (web summary)

This study reports sequencing, assembly, and characterization of the genome and transcriptome of *Wolfiporia cocos*. In addition, Three CYP450s were identified to be involved in the biosynthesis of its active component, pachymic acid.

## Introduction

*Wolfiporia cocos* is a medicinal mushroom that has been used in traditional Chinese medicine (TCM) for different therapeutic purposes for hundreds of years. It has been also used as a traditional food, namely “Indian bread”, for thousands of years by Native Americans in the USA. The sclerotium part, commonly known as Fuling in China, contains various active compounds, e.g., polysaccharides, triterpenoids, and sterols. Many of these compounds have a wide variety of pharmacological activities, such as anti-aging and immune-boosting^1–5^, anti-tumor^6,7^, and anti-inflammation^3,8–10^. Pachymic acid (PA), a lanostane-type tetracyclic triterpenoid, is one of the major medicinal compounds in *W. cocos*. Previous investigations have reported that PA has anti-inflammatory and anti-tumor activities^11,12^. Nowadays, the content of PA serves as a medicinal metabolite marker for the evaluation of Fuling quality in TCM.

Fuling has also been commonly used in numerous special food, tea, and cosmetics products because of the health benefits of PA and other compounds. As a result, its demand has increased dramatically over the past decades. To meet this demand, industries have been trying to grow fuling in the field. The required symbiosis of *W. cocos* and the roots of pine trees leads to challenges for the high production of this mushroom. Due to climate change and damage to natural habitats, it has become impossible to collect this wild mushroom from the field. Unfortunately, to chase huge commercial profits, industries have continuously cut down pine trees for large-scale cultivation on logs. This commercial activity has further caused severe deforestation of pines and destruction of the natural habitats of Fuling in China and other countries^13–15^. These problems indicate the necessity of understanding the biosynthesis of PA and for improvement of Fuling production.

**Fig. 1:**
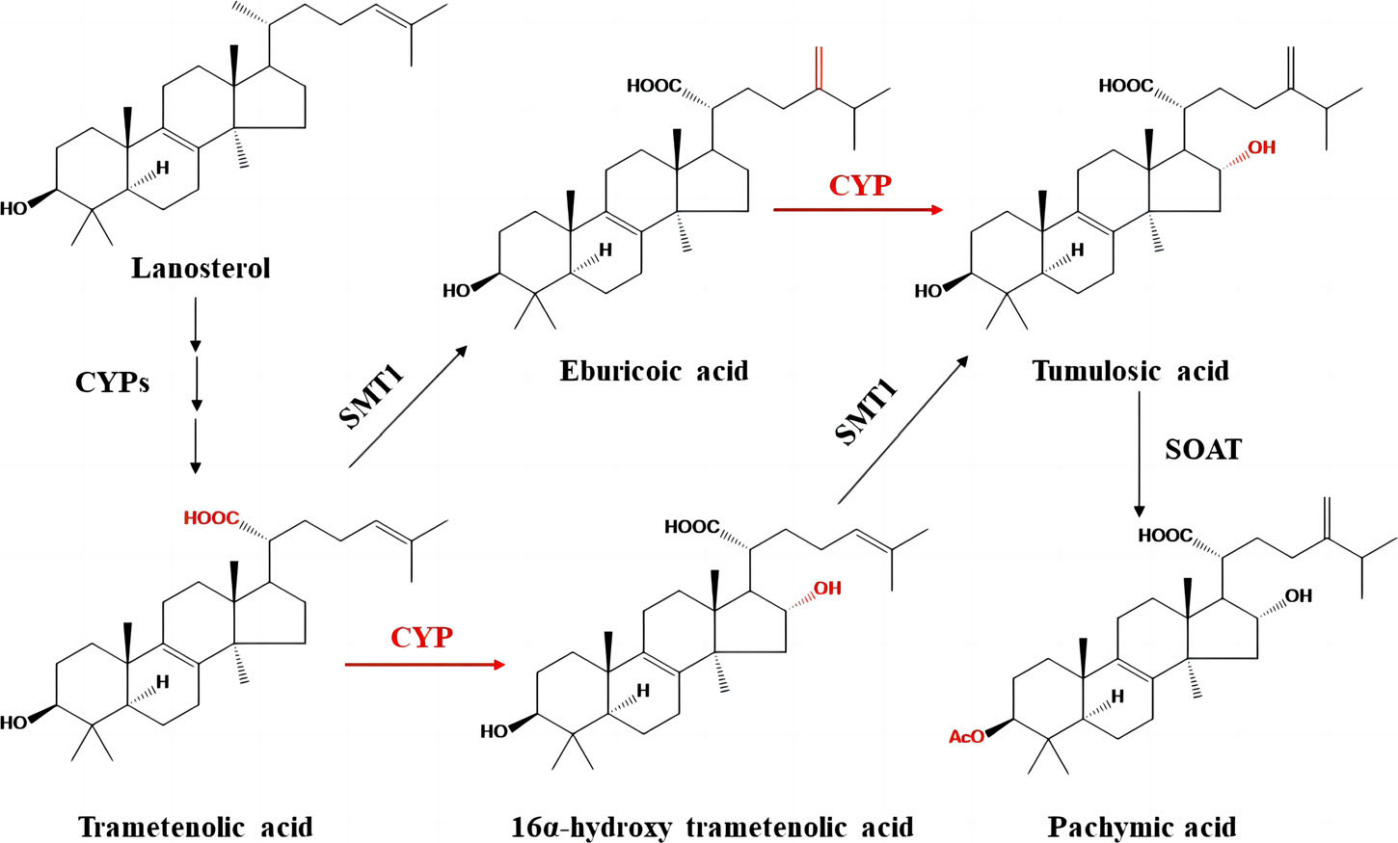
A hypothetical biosynthetic pathway of pachymic acid (PA) starting with lanosterol. CYP Cytochrome P450, SMT1 sterol C-24 methyltransferase, and SOAT sterol O-acyltransferase. CYPs highlighted in red color mean that these steps are defined in this report.

To date, the biosynthesis of PA in Fuling has remained for investigation. In our previous report, we proposed a biosynthetic pathway of PA via the mevalonate (MVA) pathway (Fig. 1)^16^. Unfortunately, genomic, transcriptomic, metabolic, biochemical, and genetic data are lacking to support this hypothesis. In this study, we reported the use of the Pacbio Sequel sequencing platform to sequence and assemble a high-quality genome of *W. cocos*. Phylogenetic analysis with the genomes of different fungi characterized a close evolutionary pedigree between *W. cocos* and *Fibroporia radiculosa*. Gene annotation characterized contracted and expanded families. The association of gene clusters with the biosynthesis of terpenoids was analyzed. Candidates of PA pathway genes were identified. Especially, three candidates of the *CYP450* family were targeted for overexpression and silencing in transgenic mycelia, in which the contents of PA and its precursors were increased and reduced, respectively. Further transcriptional analysis and metabolic profiling indicated the association of the three *CYP450* members with the biosynthesis of PA in different temperature conditions tested.

## Results

### Sequencing, assembly, annotation, and characterization of the *W. cocos* genome

We sequenced the genome of *W. cocos* (GDMCC 5.219) using the PacBio Sequel platform and obtained 28,001 Mb of data. In total, 17,742,846 subreads were assembled into 185 contigs with N50 of 1.42 Mb. The size of the final genome assembly is 58.1 Mb (Table 1). The analysis of benchmarking universal single-copy orthologue (BUSCO)^17^ was completed to evaluate the quality of the assembly. The ratio of BUSCO was 95% (Fig. S1), indicating the high quality of sequencing and assembly. Furthermore, ab initio, homology-based search, and RNA-seq were completed to predict gene models. The resulting data showed a total of 16,015 high-confidence protein-coding genes with an average length of 1,716 bp. Among the predicted protein-coding genes, 14910 (93%) genes were annotated through the NCBI NR database, 92% from the TrEMBL database, 56% from the Pfam database, 46% from the SwissProt protein database, 39% from the KOG database, 35% from the GO database, and 26% from the KEGG database. In addition, 11,575 tandem repeat sequence (TRF), 1828 simple sequence repeat (SSR), and 1139 long terminal repeat (LTR) retrotransposons sequences were detected from the *W. cocos* genome. Moreover, our annotation identified 39 ribosomal RNA (rRNA), 172 transfer RNA (tRNA), 8 small nuclear RNA (snRNA), and 5 small nucleolar RNA (snoRNA). The genomic sequences were deposited in GenBank under the Genome database (accession number JAWQPY000000000.1).

**Table 1:** A statistical summary showing differences of sequences between our sequencing and two previously reported ones of the *W. cocos* genomes

	Genome feature	GDMCC 5.219	CGMCC5.78	MD-104 SS10
Assembly quality	Assembly size (Mb)	58.1	50.6	50.5
	Total length (bp)	60,180,872	48,858,798	–
	Contig number	185	1433	1288
	Contig N50 (bp)	1,419,924	86,253	1,282,000
	Contig N90 (bp)	430,251	20,789	–
	GC content (%)	51	52	52
	Protein number	16,015	10,908	11,581
Gene annotation	GO	5652 (35%)	5263 (48%)	5831 (46%)
	Kegg	4251 (26%)	5413 (49%)	–
	KOG	6326 (39%)	5275 (48%)	6770 (53%)
	Nr	14,896 (93%)	8648 (79%)	10,730 (84%)
	Pfam	9050 (56%)	–	6156 (48%)
	Swiss prot	7383 (46%)	6070 (55%)	–
	TrEMBL	14,894 (92%)	9116 (83%)	–
	Total	14,910 (93%)	9277 (85%)	–

The absence of certain information in the original paper was denoted by a dash (“–“).

In addition, we compared our genome assembly with the previous two^15,18^(Table 1). Our assembly obtained 58.1 Mb, adding ~15% more sequences in size than the previous two. Especially, our assembly and transcriptome annotated 16,015 potential protein-coding genes, while the two previous assemblies annotated 10,908 (CGMCC 5.78) and 11,581 (MD-104 SS10) genes. Moreover, the contig numbers of our assembly are less than one-sixth of the previous two, and our assembly has longer contig N50 (bp) and contig N90 (bp) (Table 1). These results indicate that the use of new sequencing technologies can improve assembly and characterize the genome of this medicinal mushroom.

**Fig. 2:**
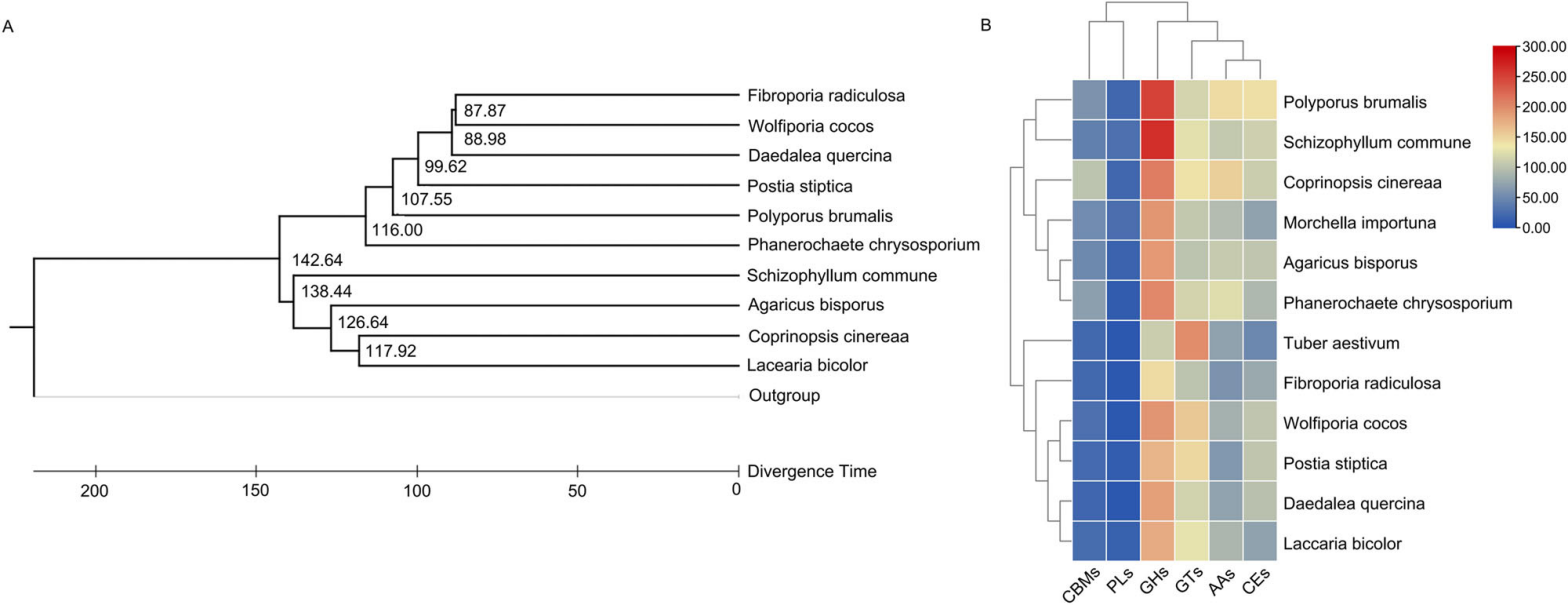
Phylogenetic and cluster analysis of *W. cocos* (Poria_cocos) and other 11 fungus species. **A** The phylogenetic tree denoted by the divergence time was constructed with amino acid sequences of 676 single copy-encoded proteins from the 12 species via the Time tree software. **B** A heatmap with clustering was built with RPKM values of six genes encoding carbohydrate-active enzymes (CAZymes) in the 12 fungus species. The heatmap indicates the phylogenetic relationship of *W. cocos, Fibroporia radiculose*, and 4 other species are clustered together.

To understand the potential evolutionary relationship among the genomes of the *W. cocos* and other fungi, we blasted the entire genome at the NR database to obtain matched species. In addition to the two previous *W. cocos* genomes, the genomes of the other five species were revealed from the GenBank (Fig. S2). However, the similarities between the genomes of *W. cocos* and the other five only ranged from 1.15% to 2.67%, indicating the uniqueness of the genome of *W. cocos*. To understand the phylogenetic relationship, we analyzed 12 fungus genomes and identified 676 single-copy homologous genes that encoded proteins (Table 2). Based on these, the amino acid sequences of the homologous proteins were used to construct a phylogenetic tree. The resulting tree clustered *W. cocos* and *Fibroporia radiculosa* (Fig. 2A). The growth of *W. cocos* mainly depends upon the degradation of the substrate, such as polysaccharides, cellulose and xylene from plants. The degradation is mainly accomplished by the carbohydrate-active enzymes (CAZymes), including carbohydrate-binding modules (CBMs), polysaccharides lyases (PLs), glycoside hydrolases (GHs), glycosyl transferases (GTs), auxiliary activities (AAs), and carbohydrate esterases (CEs). Furthermore, a heatmap was built to understand the expression profiles of *CBMs*, *PLs*, *GHs*, *GTs*, *AAs*, and *CEs* families in the 12 fungus species using the CAZymes carbohydrate databases (http://www.cazy.org/). Compared with other species, *W. cocos* expresses more genes, including *GTs* (158), *CEs* (103) and *GHs* (193). The results also revealed that *W. cocos*, *Fibroporia radiculosa*, and the other four species were in the same cluster (Fig. 2B). These phylogenetic data suggest a close phylogenetic relationship between *W. cocos* and *F. radiculosa*, which is consistent with their similar preference for growth conditions. *W. cocos* is a wood-rotting fungus. *F. radiculosa* is a brown rot fungus, and that decomposes lignin, targeting the rapid deconstruction and utilization of hemicellulose and cellulose^19,20^.

**Table 2:** Statistics of genes number in each family and contracted and expanded gene family data

Species	Multi ortholog	Other ortholog	Single ortholog	Species-specific	Contraction	Expansion
*Agaricus bisporus*	1939	5082	676	2716	4	12
*Coprinopsis cinereaa*	2034	6298	676	5200	2	22
*Daedalea quercina*	2060	6318	676	3108	6	12
*Fibroporia radiculosa*	1930	5127	676	1464	7	9
*Laccaria bicolor*	2131	6535	676	8777	2	27
*Morchella importuna*	1852	4248	676	4682	16	18
*Phanerochaete chrysosporium*	2121	6523	676	4255	6	16
*Polyporus brumalis*	2251	8211	676	7053	4	48
*W. cocos*	2730	7179	676	5356	4	53
*Postia stiptica*	2342	7609	676	3552	4	32
*Schizophyllum commune*	2141	5982	676	4197	1	21
*Tuber aestivum*	1788	3863	676	2836	19	17

To understand whether gene clusters existed in the genome of *W. cocos*, we performed sequence analysis with antismash method (antiSMASH fungal version [secondarymetabolites.org]). The resulting data revealed that 24 contig fragments had 37 gene clusters. Of these, 19 gene clusters were shown to associate with the biosynthesis of terpenoids. Main examples included genes that were annotated to encode Aldo/keto reductase family oxidoreductase, cyclase metal-binding domain, and others (Table S1).

### Liquid chromatography-mass spectrometry-based targeted metabolic profiling of PA pathway candidate metabolites

The biosynthesis pathway of PA is proposed to derive from lanosterol (Fig. 1). To understand potential intermediates, we performed liquid chromatography–mass spectrometry (LC–MS) to analyze the extracts of *W. cocos*. Based on the LC–MS data, we annotated 11 triterpenoids, which were reported previously^5^ (Supplementary Data 1). According to the fragments and accurate mass spectra profiles generated by LC–MS, trametenolic acid (TMA), eburicoic acid (EA), tumulosic acid (TA), and 16α-hydroxy-trametenolic acid (HTA) were annotated from the extracts. These data support the pathway steps proposed from lanosterol to PA (Fig. 1).

Meanwhile, according to the previous studies that the optimum temperature for the growth of mycelium was 25–30 °C^21,22^, we compared the effects of 28 °C with two unfavorable temperatures (22.5 and 33.5 °C) on the formation of PA. LC–MS analysis was performed to measure the contents of PA in the cultured mycelium of *W. cocos*. The results showed that the contents of PA were 0.19 mg/g (dry weight, DW), 1.83 mg/g (DW), and 1.30 mg/g (DW) at 22.5, 28 and 33.5 °C, respectively (Fig. S3). These results indicate that 28 °C is better than the other two temperatures (22.5 and 33.5 °C) grown in the PDA media for the biosynthesis of PA.

### Mining CYP450 family from the genome and transcriptomes

Functional annotation of the transcriptome of *W. cocos* (Swiss, NR) identified 298 genes encoding CYP450 enzymes, including 177 monooxygenases and other 121 types of reductases. The annotation via KOG identified 12 CYP families including CYP2, CYP3, CYP4, CYP5, CYP6, CYP9, CYP11, CYP12, CYP19, CYP24, CYP26, and CYP27. Furthermore, this annotation identified 159 CYP450 members that were proposed to be involved in steroid biosynthesis, benzoic acid degradation, aromatic compound degradation, and other metabolic processes (Supplementary Data 2).

**Fig. 3:**
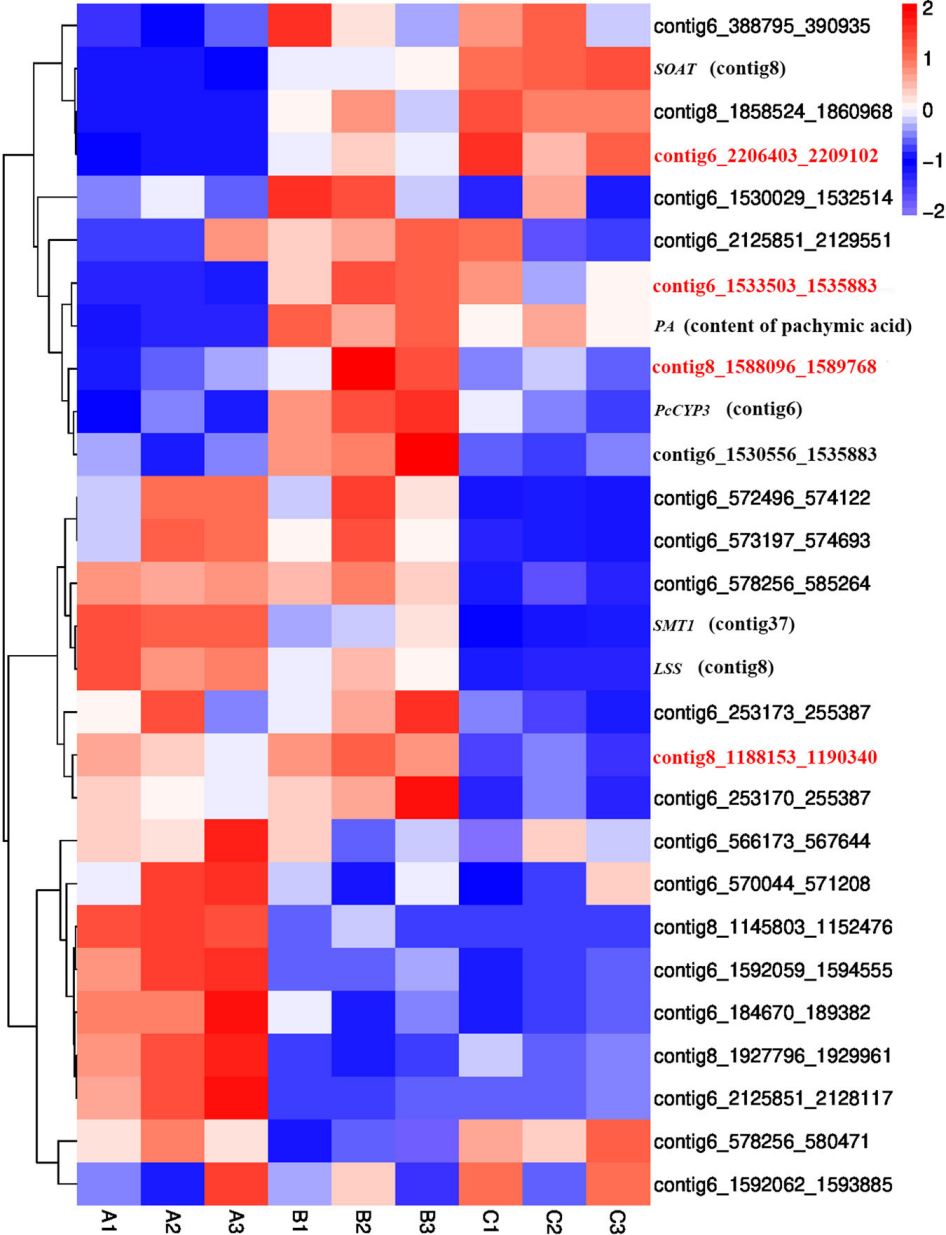
Expression patterns analysis of candidate genes potentially involved in PA biosynthesis in *W. cocos* mycelia at different temperatures. A heatmap showing the differential expression profiles of 27 genes in mycelia of *W. cocos* grown in 22.5 °C (A1–A3), 28 °C (B1–B3) and 33.5 °C (C1–C3)). A1–A3, B1–B3, and C1–C3: three biological replicates in each condition.

We further analyzed CYP450 members that were likely clustered with other genes associated with the PA biosynthesis. We completed RNA-seq of *W. cocos* mycelium cultured under 22.5, 28 and 33.5 °C conditions and assembled three transcriptomes with a total of 690 Mb in size (NCBI with accession number SRX18292007). Expression profiling analysis identified several differentially expressed genes (DEGs) in *W. cocos* under three different culture conditions (22.5, 28, 33.5 °C). Specifically, there were 940 up-regulated and 1277 down-regulated DEGs at 22.5 vs.28 °C, 1524 up-regulated DEGs and 1749 down-regulated DEGs at 22.5 vs. 33.5 °C, and 554 up-regulated DEGs and 753 down-regulated DEGs at 28 vs. 33.5 °C (Fig. S4). Moreover, KEGG and GO enrichment analyses were performed to understand the differentially expressed genes in *W. cocos*. The annotation results from the KEGG analysis showed that the DEGs were mainly enriched in the ribosome biogenesis and carbon metabolic pathways (Figs. S5–S7. S8–S10). We previously identified two genes involved in PA biosynthesis, sterol C-24 methyltransferase (SMT1) and sterol O-acetyltransferase (SOAT)^5^ (Fig. 1). Based on sequences of the genome and transcriptomes, we used the proximity of location and the similarity of expression patterns to localize potential gene clusters between CYP450 genes and *SMT1* or *SOAT*. In addition, two genes, one encoding lanosterane synthase (LSS)^4^ and the other encoding PcCYP3, that both were identified involving in the PA synthesis, were used for the cluster analysis. The resulting data showed that *LSS* and *SOAT* were in the contig 8 fragment, *SMT1* was in the contig 37 fragment, and *PcCYP3* was in the contig 6 fragment. Genomic sequence analysis revealed that 34 CYP450 genes were located in these three contig fragments (Table S2). The gene annotation and the similarity analysis of expression patterns further identified 23 *CYP450* genes. Based on the expression profiles, the 23 CYP450 genes, *LSS*, *SOAT*, *SMT1*, and *PcCYP3*, were used to develop a heatmap, which grouped these 27 genes into three clusters (Fig. 3). These data suggest that these genes are likely associated with the biosynthesis of PA (Table 3). In the first cluster, the expression patterns of *WcCYP52* (contig6_2206403_2209102), *WcCYP64-2* (contig8_1588096_1589768) and *WcCYP_FUM15* (contig6_1533503_1535883) were similar to those of *PcCYP3* and *PcSOAT* (Fig. S11). In the second cluster, *WcCYP64-1* (contig8_1188153_1190340) was located in the same contig8 fragment as *LSS* and had a similar expression pattern to that of *LSS* and *SMT1* (Fig. S11). Sequence analysis revealed the length of the four CYP450 candidates in the range of 1,000 to 2,000 bp. Furthermore, based on the annotation from the Swiss-Prot database, *WcCYP52*, *WcCYP64-2, WcCYP_FUM15*, and *WcCYP64-1* belong to CYP2, CYP4, CYP19, and CYP26 families, respectively.

**Table 3:** A list of genes proposed to associate with the pachymic acid (PA) biosynthesis

Gene name	Gene number	Expression Level		
		22.5 °C	28 °C	33.5 °C
*LSS*	contig8_71354_73949	47.39	38.39	23.86
*SMT1*	contig37_161854_163006	153.89	85.77	37.06
*SOAT*	contig8_236265_238450	57.85	90.70	126.45
*PcCYP3*	Contig 6_372163_373540	13.65	28.66	16.36
*WcCYP64-1*	contig8_1188153_1190340	27.34	3.80	2.63
*WcCYP64-2*	contig8_1588096_1589768	0.79	2.91	1.08
*WcCYP_FUM15*	contig6_1533503_1535883	7.95	18.61	14.71
*WcCYP52*	contig6_2206403_2209102	10.33	72.22	128.58

### Overexpression of *WcCYP6*4-1, *W*cC*YP52*, and *WcCYP_FUM15* increases the activity of the PA biosynthetic pathway in transgenic *W. cocos*

**Fig. 4:**
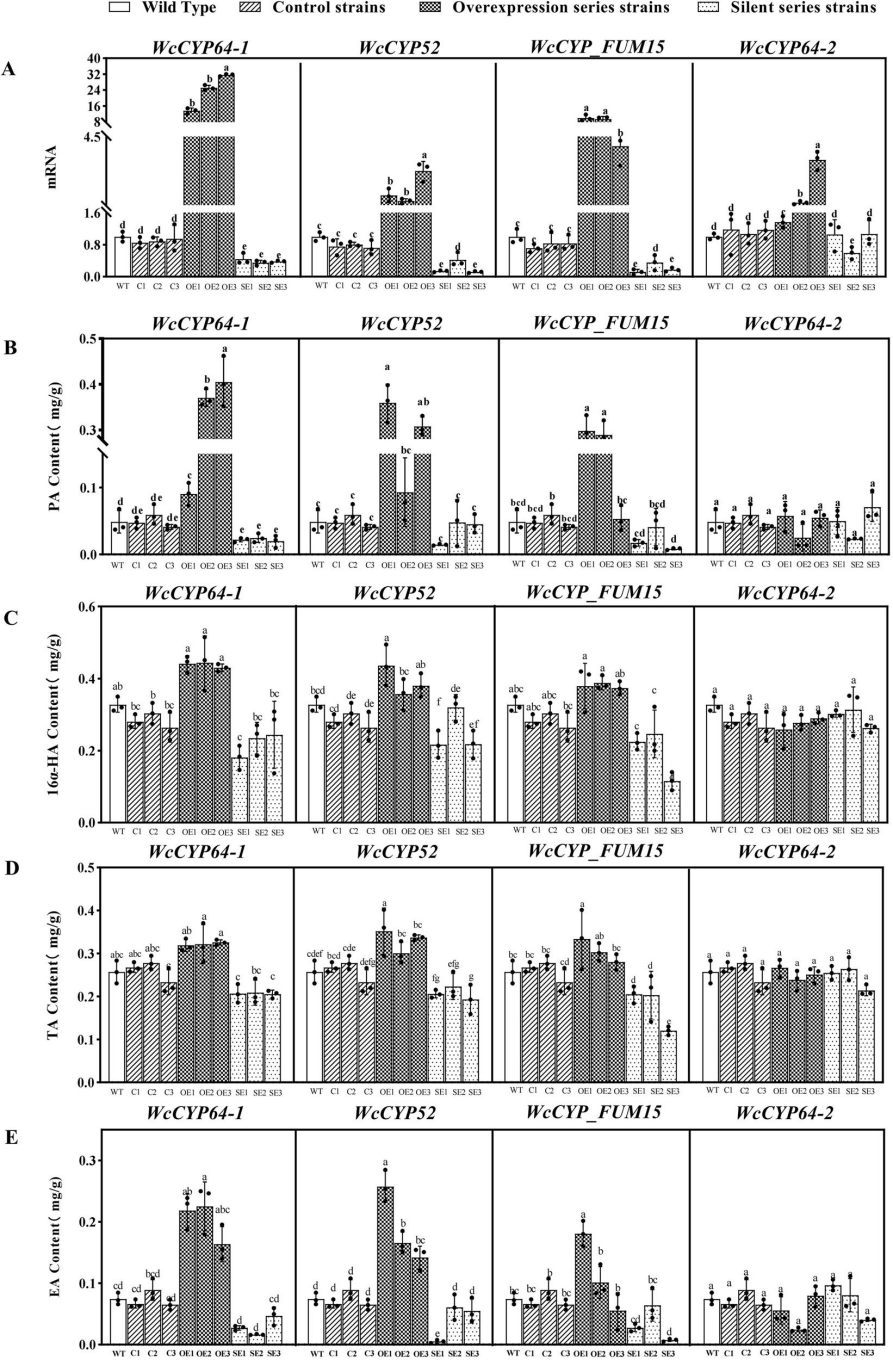
Effects of overexpression and silencing of four WcCYP64-1, WCCYP64-2, WcCYP52, and WcCYP_FUM15 on contents of pachymic acid (PA), 16α-hydroxytrametenolic acid (HA), trametenolic acid (TA), and eburicoic acid (EA). Transgenic *W. cocos* mycelia was created by either overexpression or silencing of each of four *CYP450* genes. **A** qRT-PCR analysis showed enhanced and reduced expression of each of the four genes in overexpressing levels and silencing transgenic mycelia, respectively. **B**–**E** the PA, HA, TA and EA contents in the transgenic mycelium overexpressing or silencing strains. The error bars represent SE (*n* = 3, biological replicates), and different letters denote significant differences determined by Duncan’s test at the level of *P* < 0.05.

We hypothesized that *WcCYP64-1*, *WcCYP64-2*, *WcCYP52*, and *WcCYP_FUM15* abovementioned were involved in the biosynthesis of PA and its precursors. To test their roles, these four genes were overexpressed in *W. cocos*, and three independent overexpressing lines for each gene were obtained. In addition, three empty vector transgenic lines were developed as controls. Quantitative RT-PCR analysis showed the higher expression of each gene in their transgenic lines (Fig. 4A). LC–MS assays was completed to characterize the profiles of PA, HA, EA, and TA. The contents of PA were significantly increased in two to three transgenic lines of *WcCYP64-1*, *WcCYP52* and *WcCYP_FUM15* in comparison with their wild types (Fig. 4B). Meanwhile, the contents of three precursors, TA, HA, and EA, were also significantly or slightly increased in the three transgenic lines (Fig. 4C–E). For example, the PA content in OE-1 of *WcCYP52* was 7.38 times higher than that in the wild type (Fig. 4B). In addition, the contents of HA, TA and EA in this strain were 1.33, 1.23 and 3.14 times higher than that of the wild type, respectively. The levels of these compounds were also significantly increased in the other two gene overexpressing lines. A correlation analysis showed that the expression levels of each transgene were significantly associated with the contents of PA HA, EA, and TA. For instance, the correlations between the overexpression level of *WcCYP52* and 16α-HA, EA, and TA were 0.848, 0.804, and 0.924 (*P* < 0.01), respectively. These indicated that the involvement of *WcCYP64-1*, *WcCYP52*, and *WcCYP_FUM15* in the biosynthesis of PA. By contrast, the average contents of PA and the three intermediate compounds (16α-HA, EA, and TA) in the three overexpression lines of WcCYP64-2 decreased 38.0%, 2.6%, 26.8%, and 2.9%, respectively, in comparison with those in their controls (Fig. 4). However, the differences are not significant, suggesting that *WcCYP64-2* may not be involved in the biosynthesis of PA.

Gene silencing vectors were developed to knockout *WcCYP64-1*, *WcCYP64-2*, *WcCYP52*, and *WcCYP_FUM15* in *W. cocos*. Three transgenic lines for silencing each gene, labeled as SE-1, SE-2 and SE-3, were obtained for gene expression and metabolite analysis. Quantitative RT-PCR analysis showed the reduction of each gene expression in the transgenic lines (Fig. 4A). LC–MS analysis indicated a variable reduction trend of PA, TA, HA and EA contents (Fig. 4B–E). Statistical analysis revealed that the reduction of these compound levels was slight (*P*-value higher than 0.05) or significant (*P*-value lower than 0.05) in gene silencing lines in comparison with the controls. Furthermore, a correlation analysis between the expression levels of the genes and the contents of the four compounds indicated a strong relevance (0.714 < *r* < 0.966), which depended on silencing liens. These data suggest that the full knockout of these genes is necessary to eliminate these compounds in mycelia.

## Discussion

*W. cocos* is a wood-rotting fungus species and its dried sclerotia are extensively used in numerous TCM prescriptions, special food, and other products in China. Past investigations have reported that PA, a lanosterane-type tetracyclic triterpene, is one of the main bioactive and characteristic compounds with a variety of pharmacological activities. Due to the high therapeutic demand of PA in TCM, the pharmaceutical industry has heavily harvested wild *W. cocos* from nature, and this has devastated its growth habitat and wild-type resource. In addition, although technologies for the cultivation of this fungus have been developed with pinewoods, the demand for logs has destroyed the forest of pines. This environmental destructive impact has caused ecological problems. Therefore, it is urgent to develop biotechnologies to produce PA for human health.

Although the biosynthesis of PA in *W. cocos* remains uncharacterized, we have proposed a biosynthetic pathway^5^. The proposed pathway is composed of four steps from lanosterol through TMA, EA, and TA to PA, which are catalyzed by a CYP450, SMT1, a CYP450, and SOAT, respectively. To enhance understanding of the biosynthetic pathway, we performed metabolic profiling and assembled its genome and several transcriptomes. LC–MS based profiling not only allowed identifying TMA, EA, TA, and PA, but also detected HTA, another precursor of TA. This result provided new data to elucidate the biosynthetic pathway of PA.

Our assembly developed a high-quality genome with 58.1 Mb in size, which was larger than the two previously reported (Table 1). The high quality of our assembly helped to obtain longer contigs with N50 of nearly 1.42 M bp, which contributed to annotate more genes in comparison with the two genomes previously assembled^15,18^. The additions of more sequences and annotations from our assembly are particularly useful to enhance a comprehensive understanding of the genetic information of *W. cocos*. More importantly, the combination of transcriptomes and genomic data mining allowed us to identify candidate genes that are involved in the biosynthetic pathway from lanosterol to PA. Based on these genomic data and gene cluster analysis, we not only identified the genes encoding SMT1 and SOAT but also revealed the CYP450 family. The resulting data disclosed that four *CYP450* members, *WcCYP64-1*, *WcCYP64-2*, *WcCYP52*, and *WcCYP_FUM15* clustered with *SMT1*, *SOAT*, *LSS*, and *PcCYP3* (Fig. 3), and provided evidence for a hypothesis that these genes were likely involved in the biosynthesis of PA. Further overexpression experiments indicated that *WcCYP64-1*, *WcCYP52*, and *WcCYP_FUM15* were genetically involved in the biosynthetic pathway of PA (Fig. 4). Although the activities of proteins encoded by these three genes were not tested due to the lack of substrates, based on these data, we propose that these enzymes are catalytically associated the biosynthetic steps from lansosterol to TA, from TMA to HA, and from EA to TA (Fig. 1). Several CYP450s functioning in the biosynthesis of ganoderic acid have been identified in *Ganoderma lucidum*^23,24^. Of them, CYP5139G1 and CYPFUM15A2, which catalyze 3-hydroxy-lanosta-8,24-dien-26-oic acid to 3, 28-dihydroxy-lanosta-8,24-dien-26-oic acid, belong to the same CYP450 subfamily as WcCYP_FUM15 identified in this study. However, CYP450’s involvement in PA synthesis has not been reported in fungi until now. In addition, the integration of transcriptional and metabolic profiling showed that the expression profiles of the three genes closely correspond to the formation of PA and its precursors in three tested temperature conditions. Therefore, although additional data are necessary to finally elucidate the biosynthesis of PA, these data allow us to propose enzymes that catalyze all biosynthetic steps from lansosterol to PA (Fig. 1). We anticipate that this pathway is instructional for us to use synthetic biology approaches to design and create pathways to test all reactions and produce PA and its precursors in *E. coli* or/and yeast. Taken together, the integration of multi-omics enabled us to characterize the biosynthetic pathway of PA in *W. cocos*.

Although we lacked mutants and other genetic materials of *W. cocos* to demonstrate the functions of the genes in the pathway, we could generate overexpressing and silencing lines to show the involvement of the candidates in the biosynthesis of PA. On the one hand, the gene overexpression and silencing approaches supported the function of *WcCYP64-1*, *WcCYP52* and *WcCYP_FUM15* associated with the biosynthesis of PA. On the other hand, the two approaches showed differences in altering the levels of PA and the three precursors. The overexpression of each gene significantly increased the levels of the four compounds. Unlike the significant changes resulting from the overexpression, the silencing of each of these genes led to two types of results. One was a slight reduction of these compounds, while the other was a significant reduction (Fig. 4B–E). For example, the PA contents were slightly lower in the silencing lines of *WcCYP64-1*, *WcCYP52* and *WcCYP_FUM15* than those in wild type and control transgenic lines (Fig. 4B). The reason was that although the three genes were reduced in the transcript levels, their expression was still promising in silencing lines. This result was likely associated with those slight reductions of the four compounds in the silencing lines. Therefore, the incomplete knockout of genes might allow a promising biosynthesis of these compounds. Other reasons were that the gene silencing might trigger the compensatory effects on the biosynthesis of PA because the pathway of PA might also be associated with other metabolic networks in *W. cocos*. Metabolic compensations might lead to insignificant alterations of the PA biosynthesis. Based on these data, our hypothesis is that a full knockout might block the formation of PA and its precursors. Our future work will endeavor to develop transgenic lines with a full knockout of these genes to elucidate their roles in the biosynthetic pathway of PA. In addition, we plan to express these recombinant CYP450 proteins through heterogeneous expression in *Escherichia coli* or yeast and analyze their enzymatic action in vitro to finally confirm their function. We will also determine potential compensatory metabolisms in *W. cocos* and increase the activity of the biosynthetic pathway of PA for high production.

## Methods

### Strain and cultivation condition of the *W. cocos*

The *W. cocos* wild-type strain P5.78 (species preservation number: GDMCC 5.219) used in the following experiments was obtained from the Guangdong Microbial Culture Collection Centre (Guangdong Institute of Microbiology, Guangdong Academy of Sciences). The strain was developed on potato dextrose agar (PDA) medium. Meanwhile, its suspension culture was conducted in liquid potato dextrose broth (PDB) medium contained in 250 mL E-flasks, which were placed on a shaker at a speed of 120 rpm and 28 °C. All cultures were performed in the dark condition.

### Genome sequencing and assembly

Genomic DNA was extracted from the mycelia of *W. cocos* using a modified CTAB method^25^. The resulting high-quality DNA samples were used to develop a Single Molecule Real-Time (SMRT) sequencing library using SMRTbell Express Template Prep Kit 2.0 (Pacific Biosciences) according to the manufacturer’s instructions. The resulting library was sequenced with both Illumina HiSeq X Ten (Illumina Inc., San Diego, CA, USA) and PacBio Sequel (Pacific Biosciences of California, Menlo Park, CA, USA) platforms. Raw reads were generated using SMRT LINK 5.0 software. Long reads generated by the Pacbio Sequel platform were assembled using the Flye v2.5 software. Short reads obtained from Illumina Hiseq X Ten were filtered with HTQC v1.92.310 to remove low-quality ones, and the resulting high-quality sequences were used to confirm the assembled sequences from Pacbio using the Pilon v1.23 software.

### Gene annotation

Genes were annotated using a combination of approaches of homology search, de novo prediction, and RNA-seq data. BRAKER2 pipeline was used to predict the gene model. Gene functions were annotated according to the best match of the alignments via the BLASTP search in the GenBank database in the Non-Redundant category curated by the National Center for Biotechnology Information (NCBI), TrEMBL, InterPro and Swiss-Prot protein. In addition, genes were blasted to obtain the best match alignments with an E-value threshold of 1E−5 in the database of the Kyoto Encyclopedia of Genes and Genomes (KEGG). Based on InterPro protein databases, the protein domains were inferred using PfamScan (pfamscan_version) and InterProScan. The motifs and domains within gene models were identified via the PFAM database. Gene Ontology analysis was completed with Blast2GO to obtain an ID for each gene.

### Phylogenetic analysis

A Blastp program was used for protein sequence alignment, selecting homologous protein pairs with *E*-value < 1e−5 and minimum coverage > 40% in the alignment region. The protein-encoding genes annotated from the genome were deduced to amino acid sequences. This step obtained genome-wide proteins, from which those proteins encoded by single-copy genes were identified. The PorthoMCL program^26^ was used to cluster the homologous proteins. Based on the blast and clustering results, in-house Perl scripts were performed to extract the 676 single-copy orthologous proteins from *W. cocos* and 11 other species. These sequences were used for alignment completed with the MUSCLE software. The alignment results were used for phylogenetic analysis via the MEGA software, in which the neighbor-joining (NJ) method was used to construct the phylogenetic tree with *Morchella importuna* and *Tuber aestivum* as outgroup species. The TimeTree database was used to estimate the divergence times in the phylogenetic tree.

### Gene family expansion and contraction analysis

Gene family expansion and contraction that occurred in the genome were analyzed with the CAFE program^27^ via a random birth and death model. This method allowed investigation of gene gain or loss within gene families across the phylogenetic tree. Subsequently, the conditional *P*-values were computed for each gene family. Families with a conditional *P*-value below 0.05 were classified as exhibiting an accelerated rate of gene gain or loss. Gene families with significant expansions or contractions (*P* < 0.05) were identified from key divergence nodes of species on the phylogenetic tree.

### Transcriptome sequencing and analysis of cytochrome P450 family

The optimum temperature for growing mycelia of *W. cocos* is 25–30 °C. To test the effects of temperature on gene expression, mycelia of *W. cocos* were cultured in PDB at 22.5 °C (a low temperature), 28, and 33.5 °C (a high temperature), respectively. After 7 days of culture, the mycelia were collected into liquid nitrogen to obtain experimental samples. In each temperature condition, three biological samples were prepared as replicates. Each biological sample was divided into two parts. One was used to perform RNA-Seq. The other was used to measure PA contents described below. Total RNA was extracted using Trizol according to the manufacturer’s protocol (Invitrogen, CA, USA), and then used to isolate mRNA with Oligo(dT)-attached magnetic beads (Illumina, San Diego, CA, USA). The mRNA was used as the template for reverse transcription to synthesize the first strand of cDNA using the Clonetech SMARTerPCR cDNA Synthesis Kit. The resulting cDNA libraries were used for RNA-Seq on an Illumina Hiseq2500 instrument. The raw sequences were initially filtered using the fastp^28^ with default parameters. Subsequently, the cleaned sequences were aligned to the reference genome using the HISAT2^29^. Quantification was completed using the cufflinks and the htseq programs, and the values were inputted into DESeq2^30^ (version 1.22.2) for differential expression analysis. To characterize differentially expressed genes (DEGs) in samples among the three temperature conditions, the threshold of false discovery rate ((FDR) was <0.05, and the log2FC (fold change) value was >1 or <−1. The differential expression levels were denoted with Reads Per Kilobase per Million mapped reads (RPKM). Based on RPKM, DEGs associated with the three temperature treatments were obtained to understand their functions and then categorized into different gene families. All transcriptomes were deposited at NCBI with an accession number of SRX18292007.

The transcriptomes were mined to identify members of the CYP450 family. In addition, to understand the effects of temperatures on the expression of *CYP450* members, three treatments were tested to identify DEGs. Next, the DEGs of *CYP450* members resulted from the treatments of three temperature conditions that were particularly selected for heatmap and clustering analysis with ImageGP (http://www.ehbio.com/Cloud_Platform/front/) with row scaling. Those DEGs of CYP450 members annotated as monooxygenases and associated with secondary metabolism were intensively examined to predict their potential involvement in the biosynthesis of PA.

### Cluster analysis of *CYP450* and the known PA pathway genes

The *CYP450* gene family and the known PA pathway genes identified from transcriptomes and genomes assembled above were used for the cluster analysis. Two principles of gene clustering, proximity in location and similar expression patterns, were used for this analysis. The algorithm of antiSMASH with the default parameters (http://antismash.secondarymetabolites.org/) was used for clustering. General Feature Format (GFF) annotation files were recorded to determine the locations of potential gene clusters. The resulting CYP450 candidate genes were then selected for correlation analysis between expression profiles and PA contents.

### Functional analysis of WcCYP64-1, WcCYP64-2, WcCYP52 and WcCYP_FUM15 in *W. cocos*

To understand the function of *WcCYP64-1*, *WcCYP64-2*, *WcCYP52*, and *WcCYP_FUM15*, we overexpressed and knocked out each of them in *W. cocos*. In brief, as reported previously ^5^, each was cloned into the pHygKS vector under the control of the Pgpd promoter. Three constructs were developed for overexpression, and three others were established for gene silencing. All constructs were introduced into *E. coli* and positive colonies were selected on a medium supplemented with ampicillin antibiotics. In addition, the transgenic lines carrying the empty pHygKS vector were used as negative controls. At least three antibiotics-resistant positive mycelia strains for each gene were obtained. Quantitative RT-PCR analysis was completed to confirm the presence of transgenes and their expression levels. The primer pairs used for expression analysis are included in Table S3. The relative expression levels were calculated using the 2^−ΔΔCT^ approach with *his* as the reference gene. Finally, three transgenic lines for each gene, three transgenic lines for empty vector, and wild-type mycelia were used for metabolite analysis described below.

### Extraction of the total triterpenes for metabolic profiling

After 5 days of growth in PDB at 28 °C, mycelia were harvested as described above. One part was stored at −80 °C for gene expression analysis. The other part was dried at 60 °C and used to exact the total triterpenes. Dry mycelia were ground into fine powder, and a 0.2 g sample was weighed into a 50-mL flask and suspended in 10 mL methanol. The flask was placed in an ultrasonic water bath for 20 min sonication at a frequency of 90 kHz and repeated two times. The mixture was filtered to a new flask. The methanol extract was further filtered through a 0.22 μm to remove debris. The resulting extract was added with methanol to adjust the final volume to 10 mL for the LC–MS analysis described below.

### Quantitation of PA content in temperature-treated *W. cocos* through HPLC

Mycelia collected from the three different temperature conditions were dried at 60 °C and ground into fine powder. 100 mg of the powdered sample was placed in a 50-mL E-flask and suspended in 10 mL methanol extraction solvent. The mixture was then sonicated in an ultrasonic water bath (90 kHz) three times each for 20 min and then placed at room temperature for 24 h. The mixture was centrifuged at 4000 rpm for 10 min. The supernatant containing PA was transferred to a new rotary glass flask, which was placed in a rotary evaporator to remove solvent to obtain dry residues at 70 °C. The remained residue was suspended in methanol. The final volume was adjusted to 5 mL. Finally, the methanol extracts were filtered through a 0.22 μm membrane to remove debris for HPLC analysis.

PA content was measured using the Agilent 1260 Infinity II HPLC system. In brief, samples were separated in a Hypersi BDS C18 column (5 μm, 250 mm × 4.6 mm, Thermo Fisher Scientific, Shanghai, China) at 30 °C. The mobile phase consisting of acetonitrile (A) and 0.1% phosphoric acid (B) was used to elute metabolites with a linear gradient as follows: 0–8 min, 80–90% A; 8–15 min, 90–95% A; 15–20 min, 95% A. The flow rate and injection volume were 1.0 mL/min and 20 μL. PA in extracts was recorded with 210 nm. PA standard was used as a positive control and developed a standard curve to calculate PA contents.

The Waters Acquity UPLC system coupled with Xevo G2xs Q-TOF MS facilitated with an ESI source (Waters, MA, USA) was used to analyze PA, HA, EA, and TA. An Acquity UPLC BEH C18 column (100 mm × 2.1 mm, particle size 1.7 μm, Waters, USA) placed at 45 °C was used to separate metabolites. The flow rate was 0.4 mL/min. The mobile phase was composed of water with 0.1% formic acid (A) and 100% acetonitrile (B). A linear gradient program was used for the elution of metabolites as follows: 0 min, 1% B; 1 min, 1% B; 15 min, 99% B; 17 min, 99% B; 17.1 min, 1% B; 20 min, 1% B. One microliter of methanol extract was injected into the column. The negative ion mode was used to ionize metabolites. MS acquisition was scanned from *m*/*z* 100–1200. The system parameters used for ionization of metabolites included capillary voltage 2000 V, cone voltage 30 V, source offset 60 V, source temperature 120 °C, dissociation temperature 450 °C, dissociation gas flow 800 L/h, and cone gas flow 50 L/h. The dissociation gas was N_2_, and the collision gas was Ar. The accurate mass and composition of the precursor and fragmentations were calculated and sequenced using the MassLynx4.1 software (Waters Corp., Milford, MA).

### Statistics and reproducibility

Three biological replicates for each treatment were set in the experiments, and strains from each replicate were sampled to sequence or measure the metabolites. Data from all the replicates was employed for the statistical analysis using the software Statistical Product and Service Solutions (SPSS 26.0). Multiple comparisons were conducted using Duncan’s test with the significance threshold of *P* < 0.05.

### Reporting summary

Further information on research design is available in the Nature Portfolio Reporting Summary linked to this article.

### Supplementary information

Supplementary Information

Description of Additional Supplementary Files

Supplementary Data 1-2

Supplementary Data 3

Reporting summary

## Data Availability

The genome data in this study have been deposited to the NCBI Genome database (accession number JAWQPY000000000.1). The transcriptome data in this study have been deposited to the NCBI SRA database (accession number SRX18292007). Furthermore, the functional annotations of AwCYP450 have been submitted to figshare (10.6084/m9.figshare.23564484). The source data for Supplementary figures and tables are available in Supplementary Data 1–3, respectively.
