# Supplementary material for: An omics-based characterization of Wolfiporia cocos reveals three CYP450 members involved in the biosynthetic pathway of pachymic acid
Source: Commun Biol. 2024 May 30;7:666. doi: 10.1038/s42003-024-06323-1 (PMC11139888; doi:10.1038/s42003-024-06323-1)
Supplement: Supplementary file 1 — Supplementary Information [file 42003_2024_6323_MOESM1_ESM.pdf]

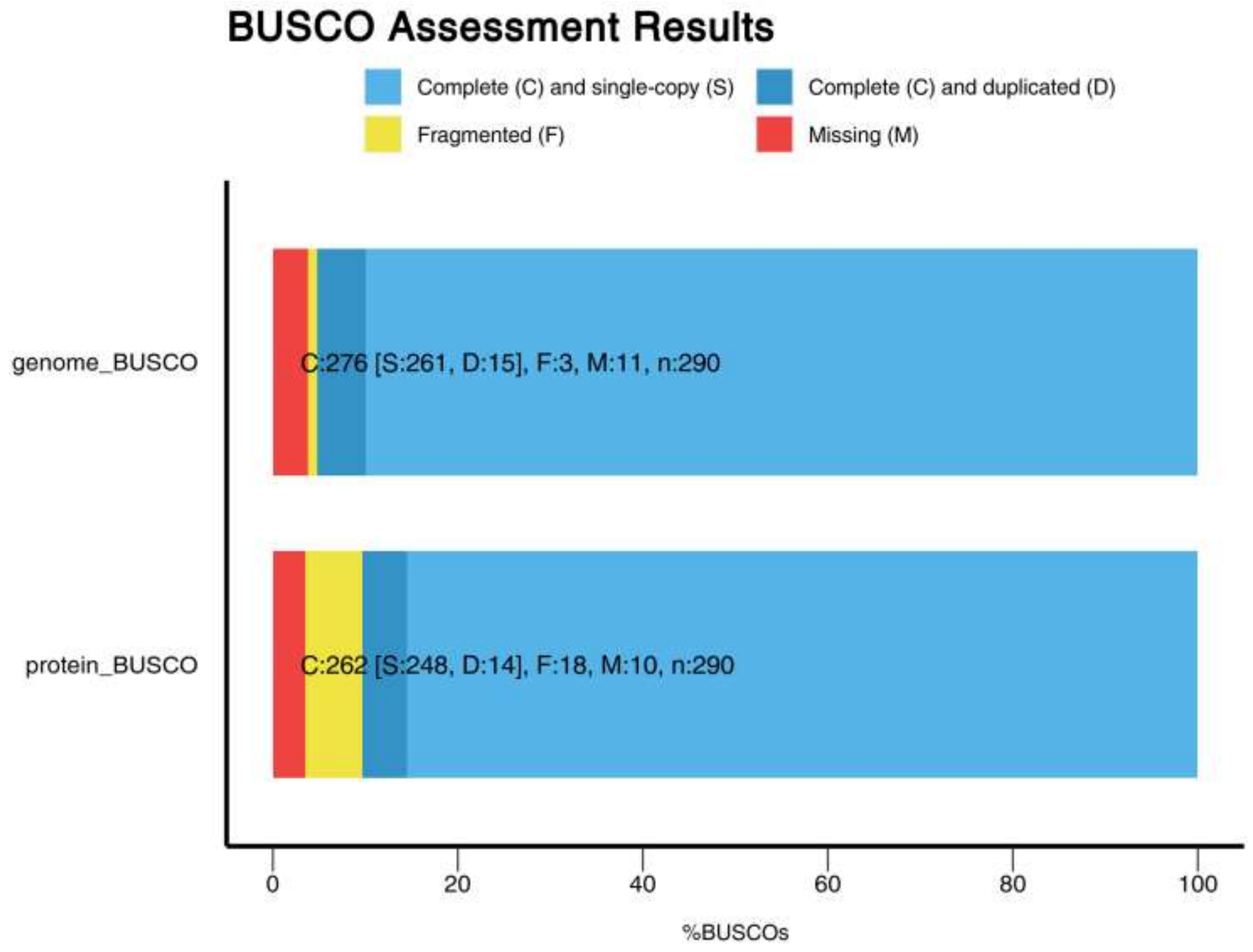

Figure. S1 Benchmarking universal single-copy orthologue (BUSCO, version 3.1.0) analysis results of the genomic sequence of *W. cocos*

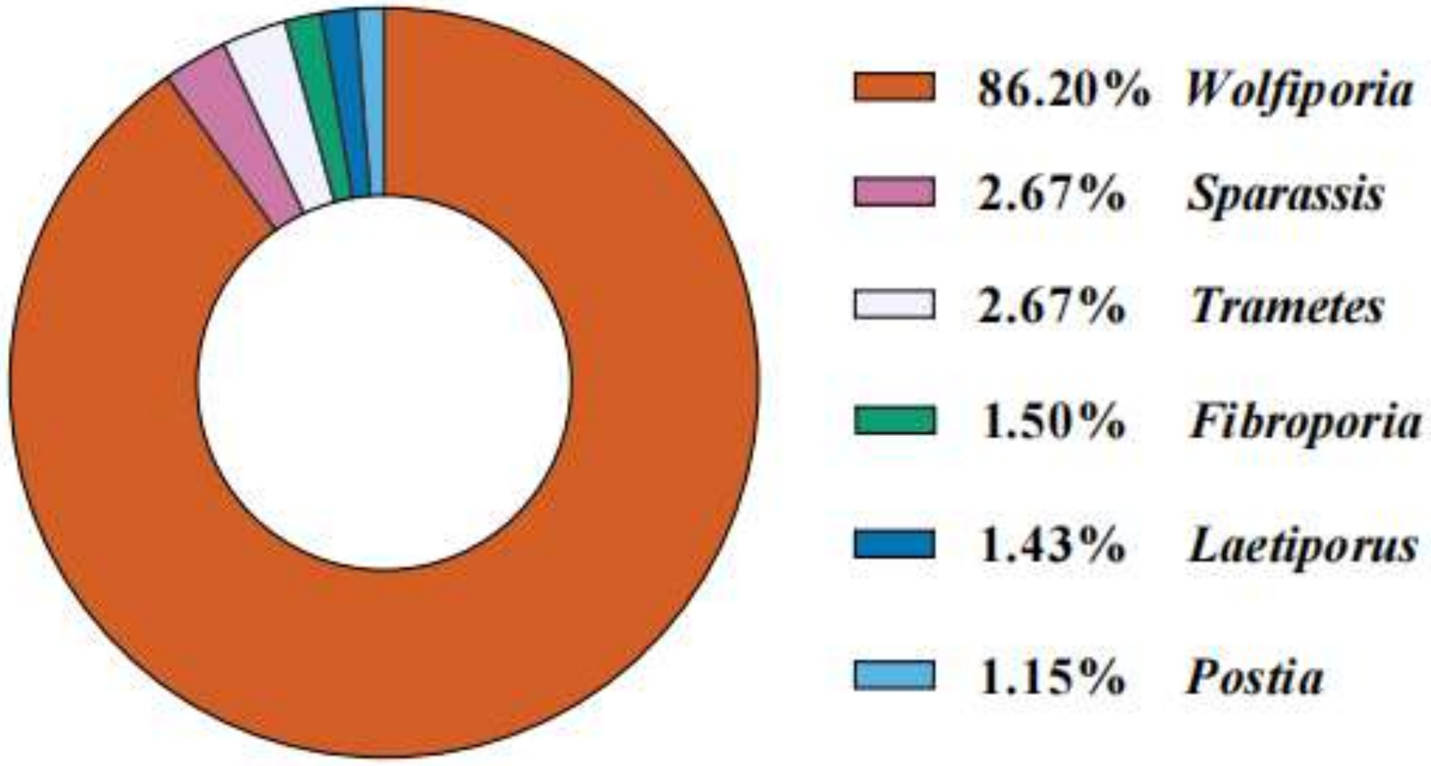

Figure. S2 A distribution pattern showing five matched species created with the NR database

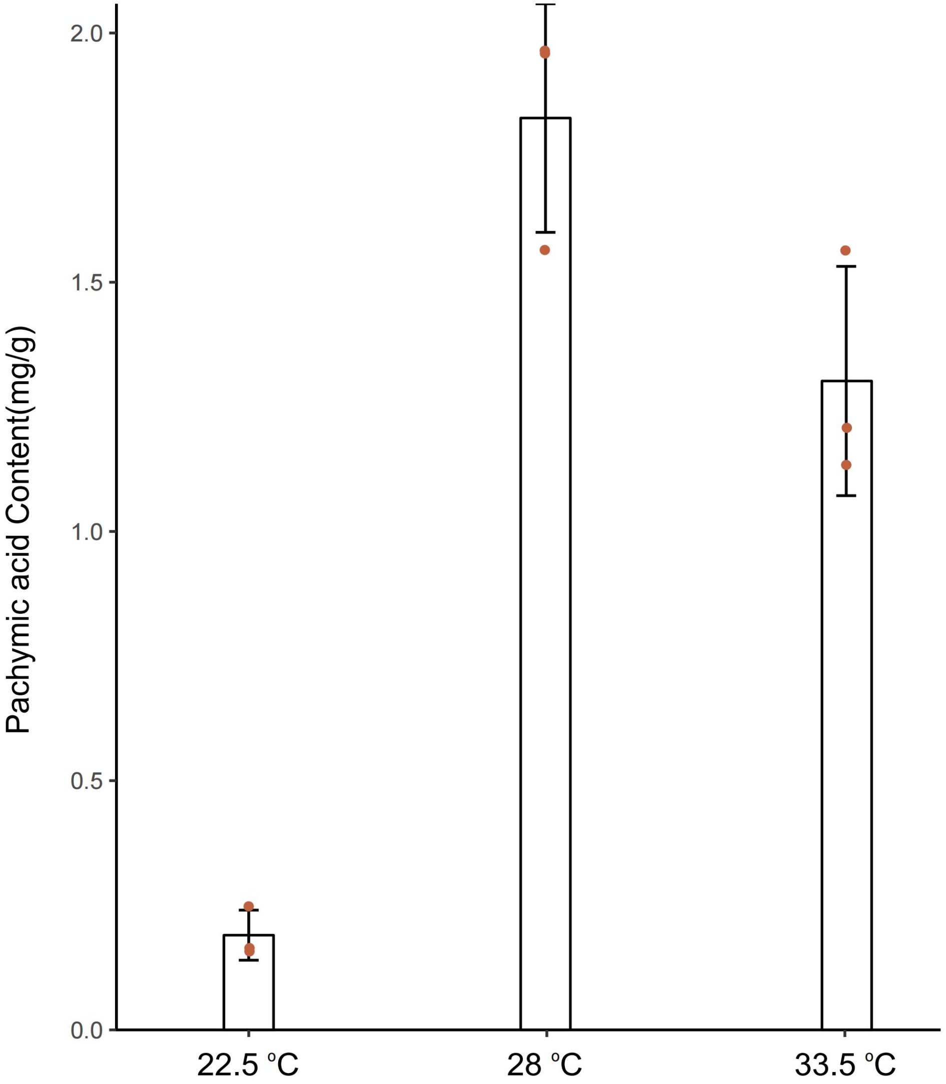

Figure. S3 Effects of three different cultural temperatures on contents of pachymic acid in mycelia of *W. cocos*. A, B and C are cultural temperatures at 22.5°C, 28°C and 33.5°C, respectively. The error bars represent SD (n=3, biological replicates), and different letters denote significant differences determined by Duncan's test at the level of $P<0.05$.

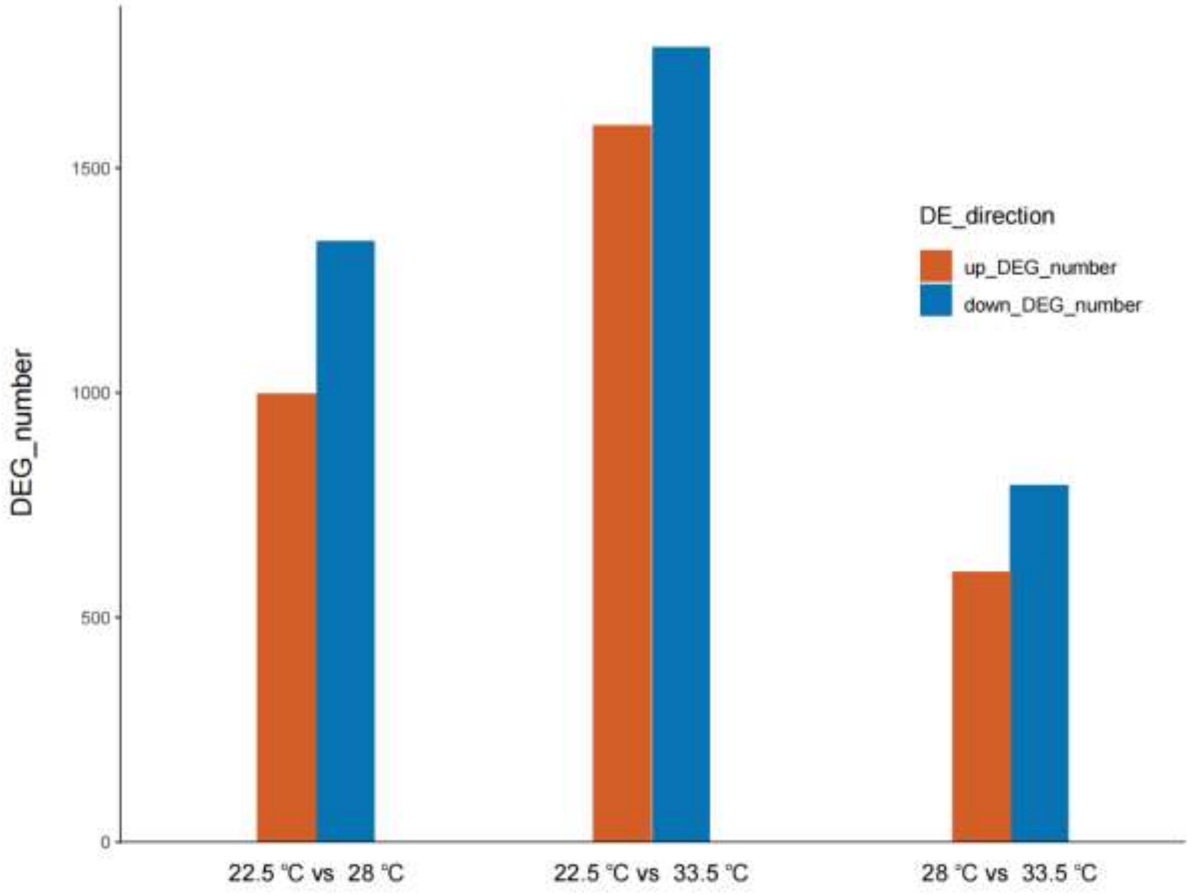

Figure. S4 Effects of three different culture temperatures on gene expression of W. cocos via comparative transcriptome analysis. Transcriptomes assembled from three temperatures (22.5°C, 28°C and 33.5°C) were compared to obtain deferentially expressed genes (DEGs) including both up-regulated and down-regulated ones.

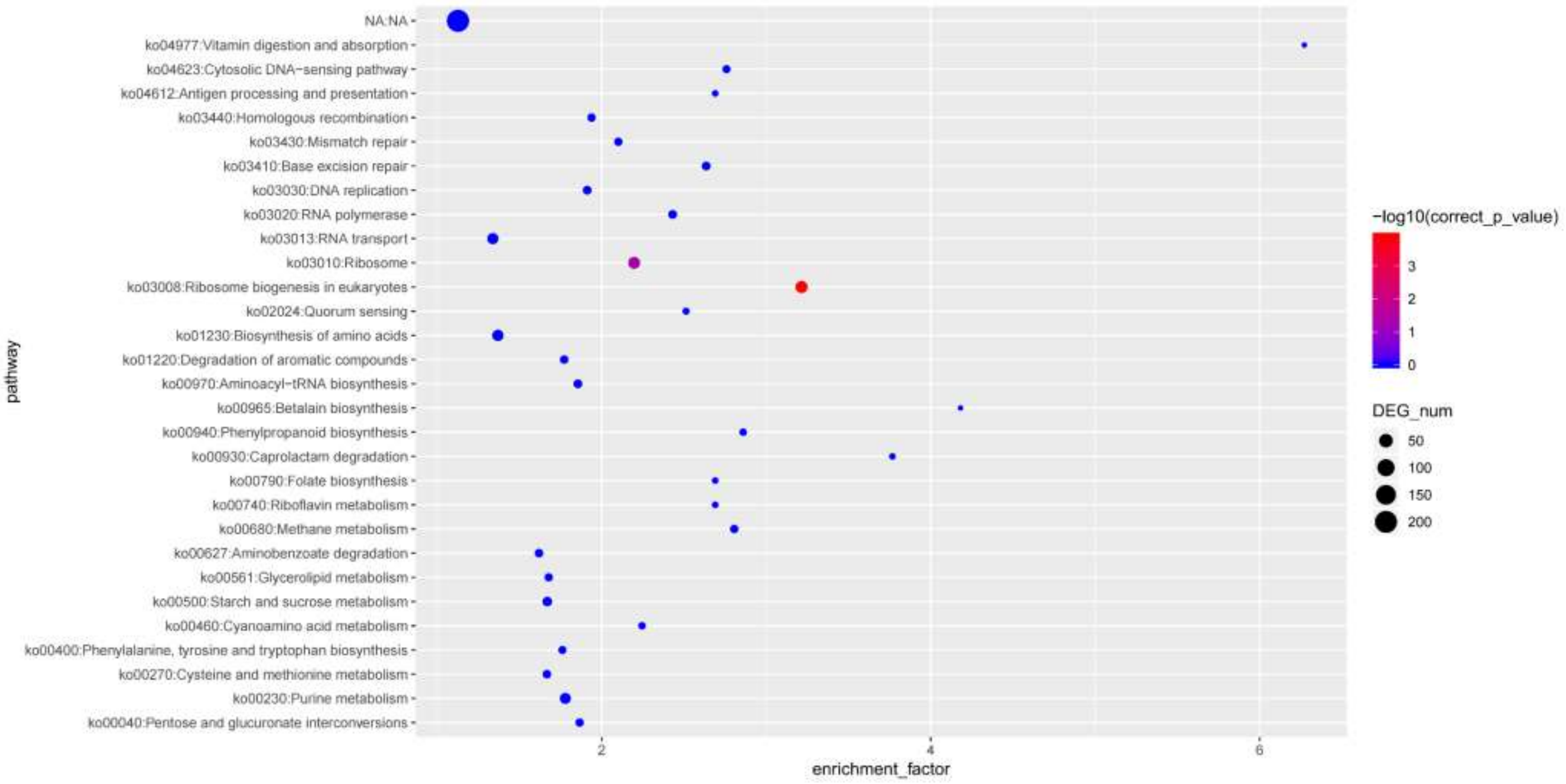

Figure. S5 KEGG enrichment results of different expression genes between the samples of 22.5°C and 28°C

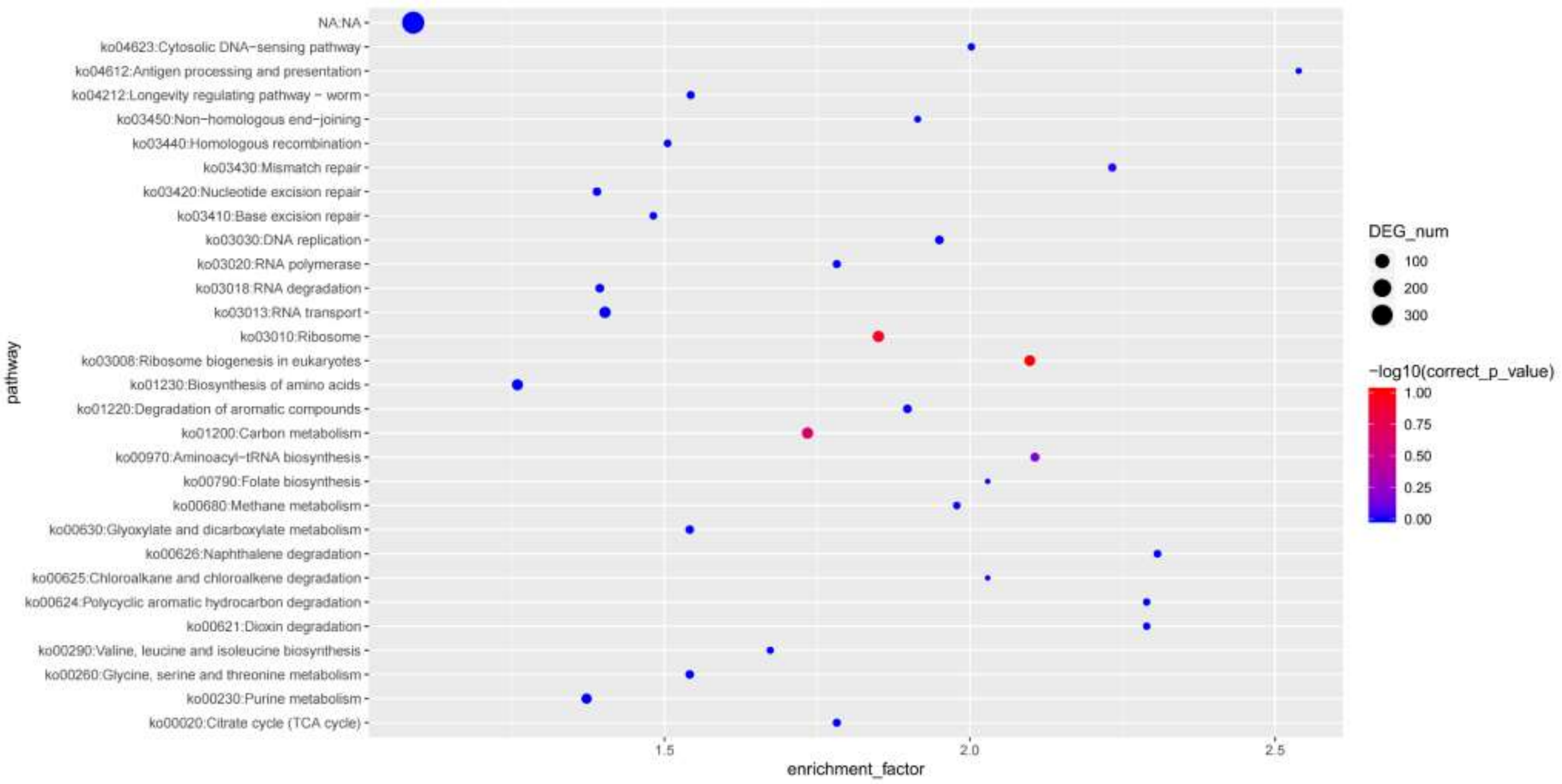

Figure. S6 KEGG enrichment results of different expression genes between the samples of 22.5°C and 33.5°C

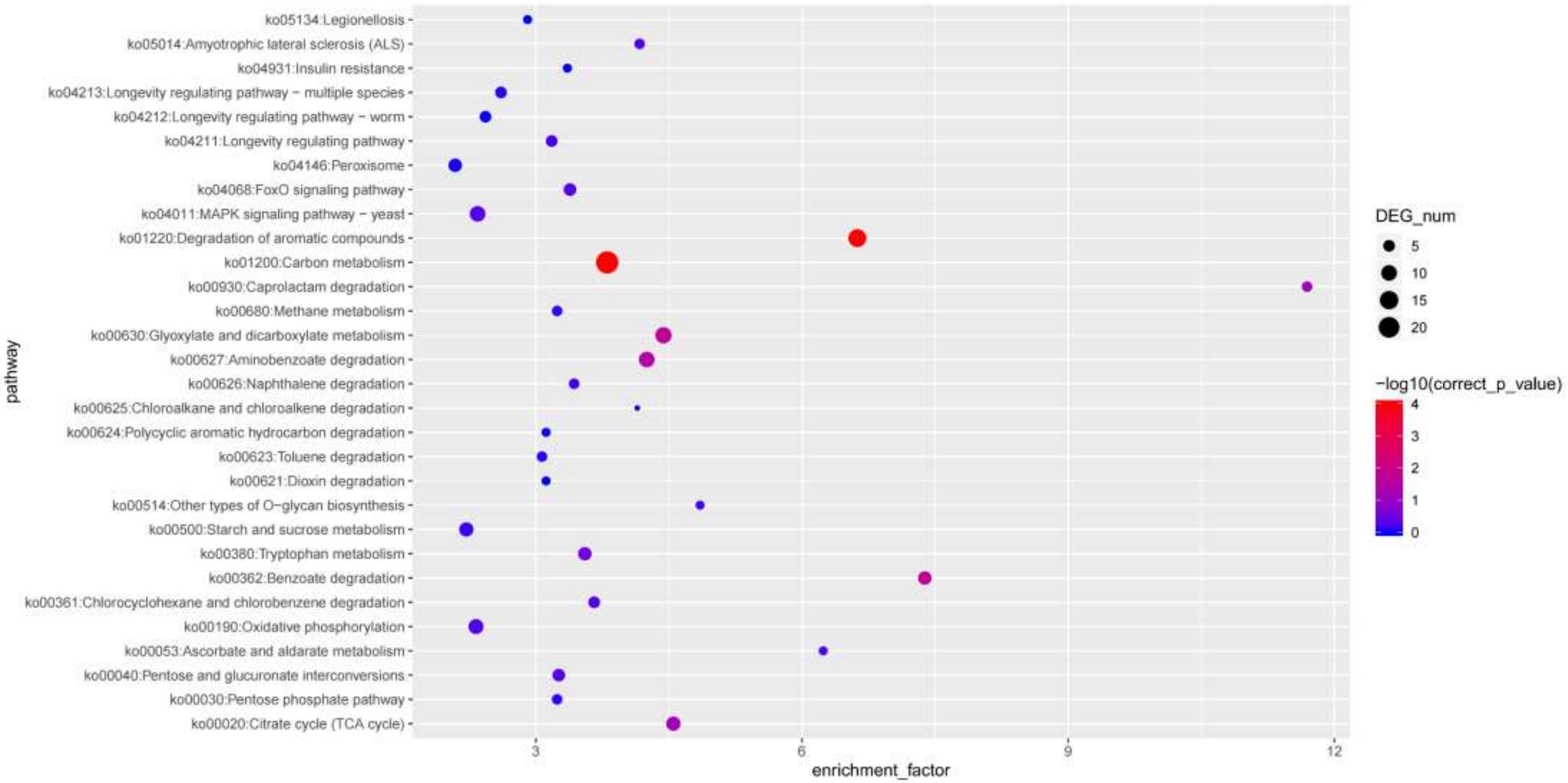

Figure. S7 KEGG enrichment results of different expression genes between the samples of 28°C and 33.5°C

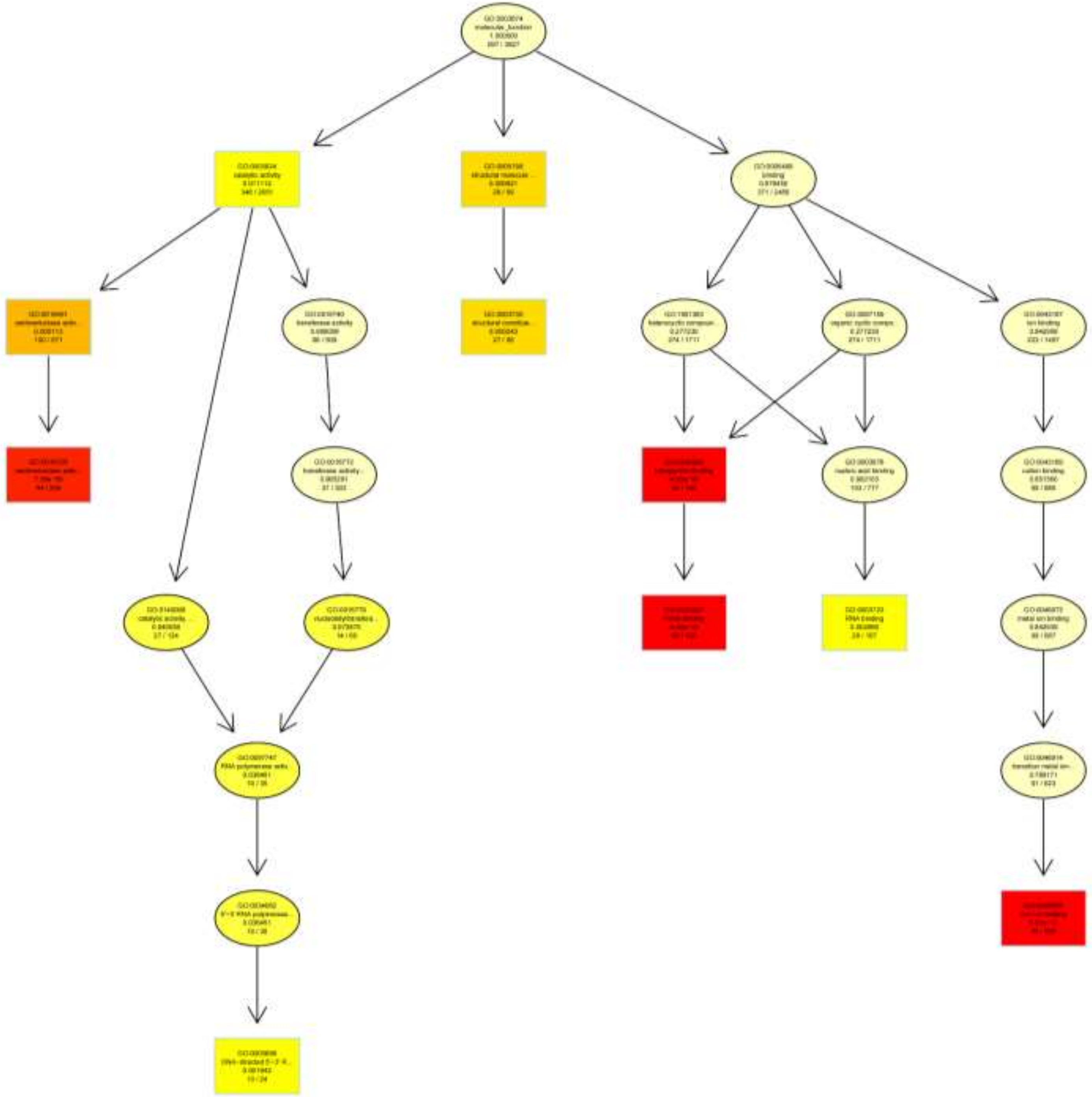

Figure. S8 MF_topGO enrichment results of different expression genes between the samples of 22.5°C and 28°C

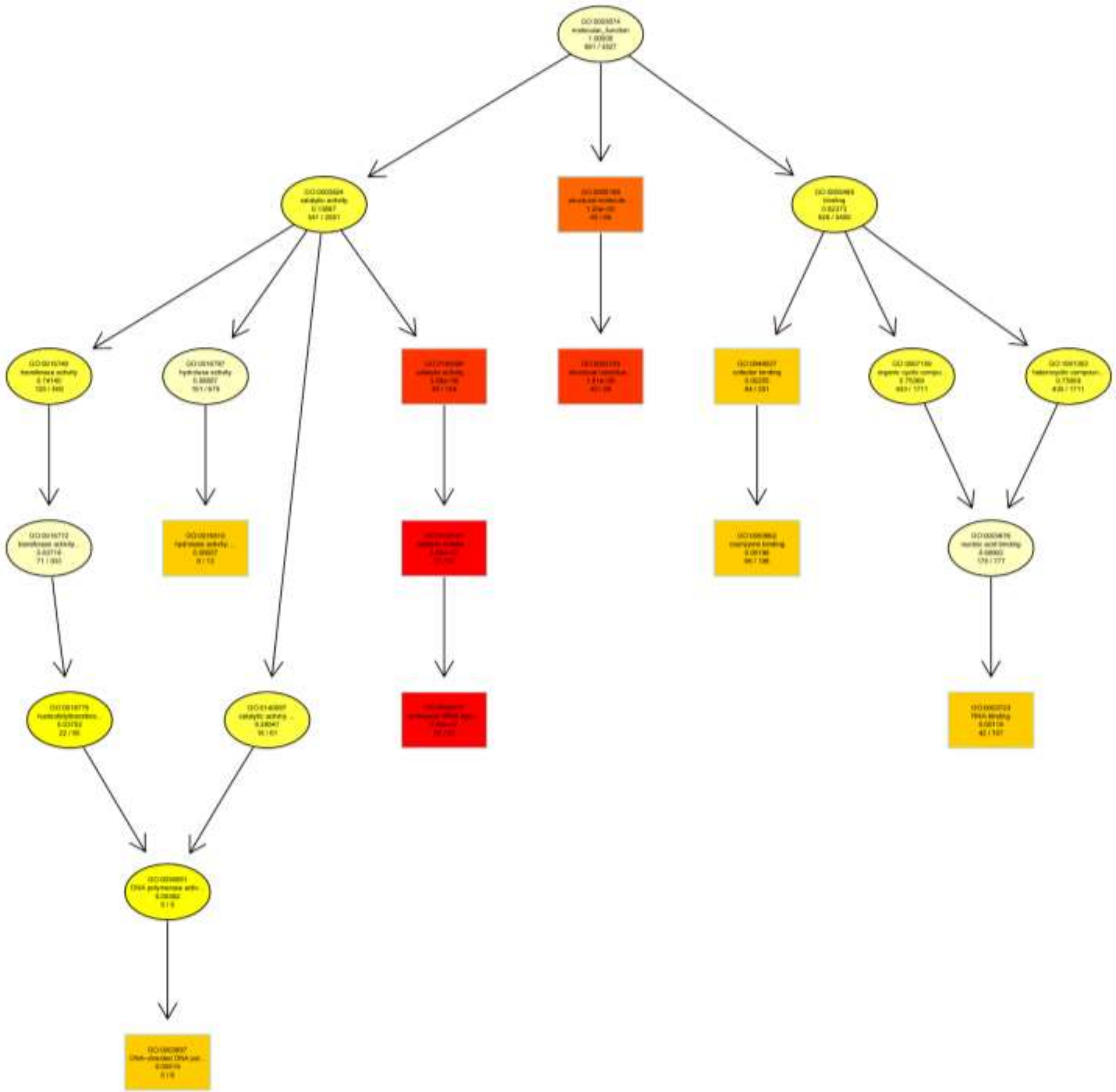

Figure. S9 MF_topGO enrichment results of different expression genes between the samples of 22.5°C and 33.5°C

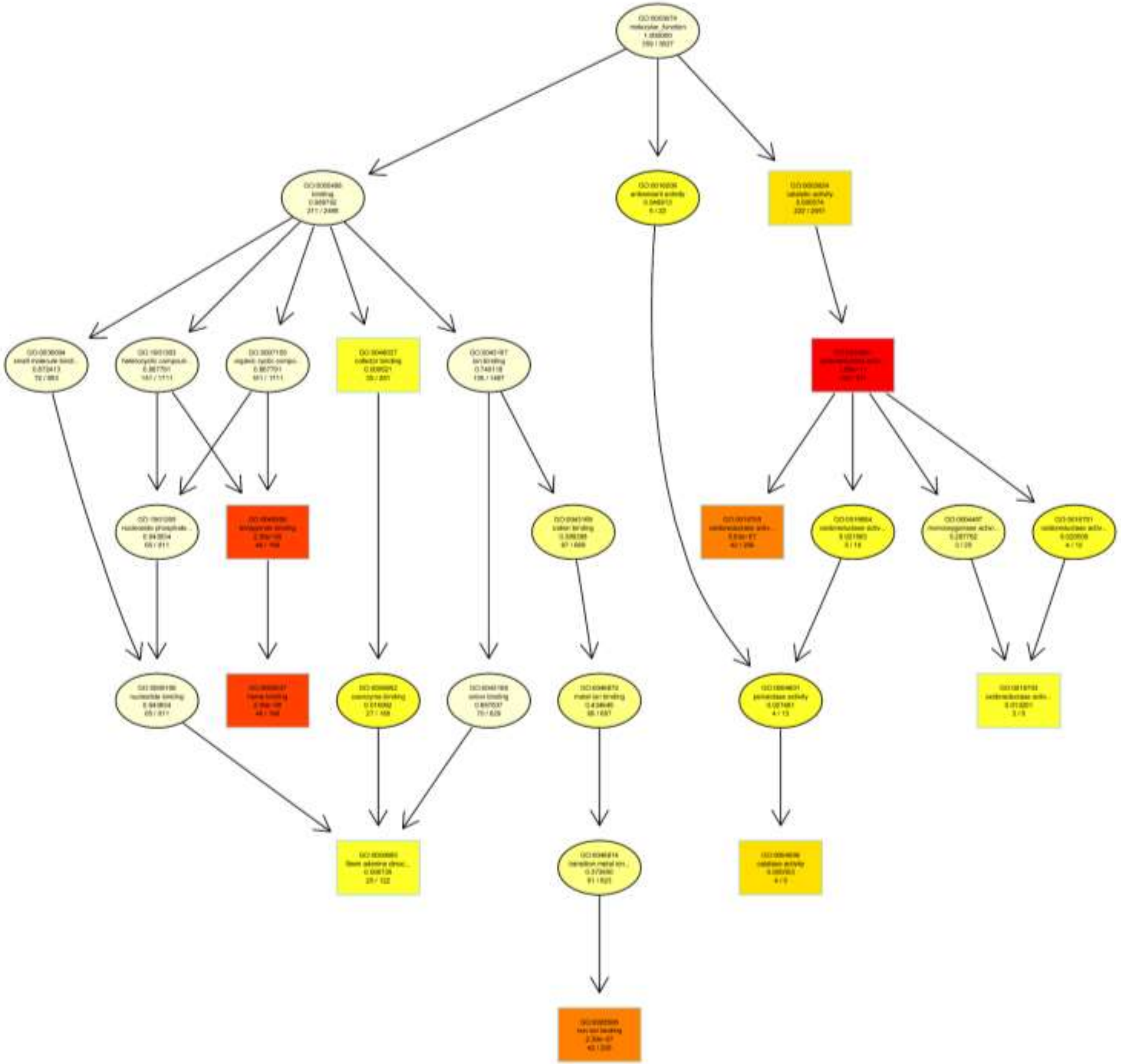

Figure. S10 MF_topGO enrichment results of different expression genes between the samples of 28°C and 33.5°C

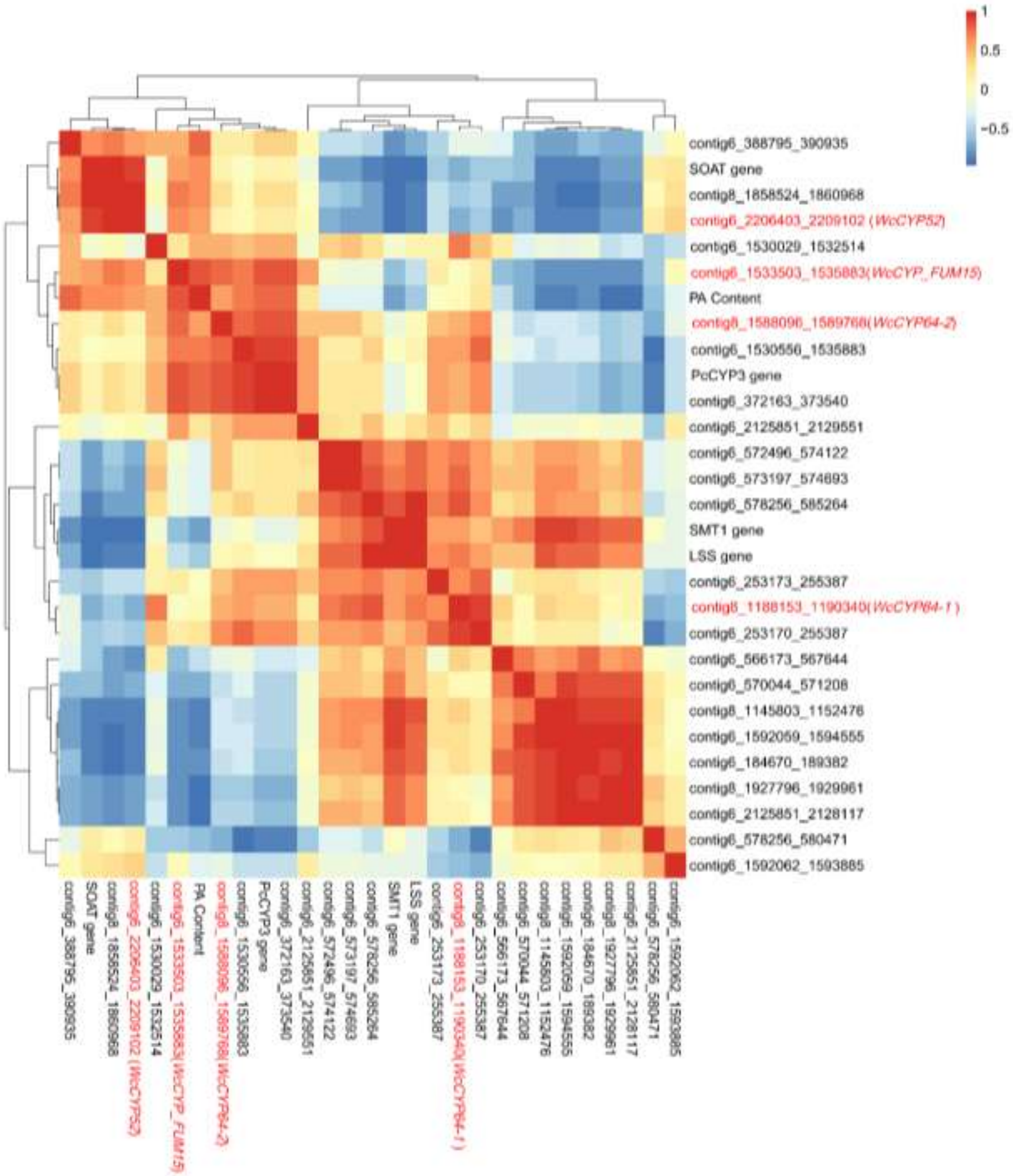

Figure. S11 A heatmap showing the correlation of expression patterns between *WcCYP450* and *WcLSS*, *WcSMT1*, and *WcSOAT*

Table S1 Analysis of gene clusters associated with secondary metabolisms in the genome of *W.cocos*

Region	Type	From	To	biosynthetic pathway
Region 1.1	terpene	412,010	454,353	terpene: TRI5
Region 1.2	NRPS-like	646,699	691,721	
Region 1.3	terpene	931,894	950,504	terpene: TRI5
Region 1.4	terpene	1,551,552	1,572,602	terpene: TRI5
Region 1.5	NRPS-like	1,854,240	1,902,276	
Region 1.6	T1PKS	2,428,804	2,478,060	
Region 2.1	terpene	1,502,155	1,526,517	terpene: phytoene_synt
Region 2.2	indole	2,875,699	2,897,242	
Region 2.3	terpene	3,056,649	3,077,743	terpene:TRIS5； MCOG1038:phenylalanine-specific permease； SMCOG1001:short-chain dehydrogenase/reductase SDR
Region 3.1	terpene	292,754	314,076	terpene: Terpene_synth_C； SMCOG1052:Terpene synthase/cyclase metal-binding domain protein
Region 5.1	T1PKS	580,774	630,572	
Region 7.1	terpene	863,639	885,676	terpene: phytoene_synt
Region 8.1	NRPS-like	688,141	735,652	
Region 9.1	NRPS-like	409,332	450,852	
Region 9.2	NRPS-like	800,034	843,984	
Region 10.1	terpene	79,457	99,142	PF00561； SMCOG1039:aldo/keto reductase family oxidoreductase； terpene: Terpene_synth_C； SMCOG1052:Terpene synthase/cyclase metal-binding domain protein
Region 10.2	terpene	155,470	177,113	terpene: Terpene_synth_C； SMCOG1052:Terpene synthase/cyclase metal-binding domain protein
Region 10.3	NRPS-like	326,131	418,316	
Region 10.4	terpene	456,041	473,830	terpene: Terpene_synth_C； SMCOG1052:Terpene synthase/cyclase metal-binding domain protein
Region 10.5	terpene	511,715	552,430	terpene: Terpene_synth_C； SMCOG1052:Terpene synthase/cyclase metal-binding domain protein； RmlD_sub_bind； SMCOG1010:NAD-dependent epimerase/dehydratase ； HMGL-like； SMCOG1271:2-isopropylmalate synthase； PF00561
Region 11.1	terpene	641,277	662,623	Terpene_synth_C； SMCOG1052:Terpene synthase/cyclase metal-binding domain protein ； Pkinase； SMCOG1030:serine/threonine protein kinase
Region 14.1	T1PKS	95,979	144,456	
Region 15.1	terpene	98,392	119,649	Pkinase； SMCOG1030:serine/threonine protein kinase； Terpene_synth_C； SMCOG1052:Terpene synthase/cyclase metal-binding domain protein； p450； SMCOG1034:cytochrome P450
Region 16.1	T1PKS	848,247	891,326	
Region 17.1	terpene	488,423	509,116	terpene: TRI5； SMCOG1038:phenylalanine-specific permease
Region 19.1	terpene	683,889	702,424	Abhydrolase_6； PF00561； SMCOG1262:haloalkane dehalogenase； Terpene_synth_C； SMCOG1052:Terpene synthase/cyclase metal-binding domain protein
Region 19.2	terpene	785,518	818,954	Terpene_synth_C； SMCOG1052:Terpene synthase/cyclase metal-binding domain protein ；
Region 21.1	T1PKS	611,508	660,059	
Region 30.1	T1PKS,NRPS-like	338,866	400,985	
Region 32.1	terpene	38,237	59,455	terpene: TRI5
Region 36.1	terpene	339,397	360,703	Terpene_synth_C； SMCOG1052:Terpene synthase/cyclase metal-binding domain protein
Region 41.1	terpene	170,959	192,191	terpene: TRI5
Region 43.1	NRPS-like	97,070	141,267	
Region 45.1	NRPS-like	298,026	342,028	
Region 58.1	NRPS-like	105,915	149,915	
Region 111.1	T1PKS	3,624	50,132	
Region 147.1	NRPS-like	1	13,120	

Table S2 A summary of genes encoding CYP450 located in the same contigs

No.	Contig6	contig8	Contig 37
1	contig6_573197_574693	contig8_1145803_1152476	contig37_260566_263266
2	contig6_572496_574122	contig8_1188153_1190340	contig37_261085_263263
3	contig6_578256_585264	contig8_1588096_1589768	
4	contig6_566173_567644	contig8_1794118_1796124	
5	contig6_372163_373540	contig8_1801676_1803717	
6	contig6_184670_189382	contig8_1858524_1860968	
7	contig6_1592059_1594555	contig8_1927796_1929409	
8	contig6_253173_255387	contig8_1927796_1929961	
9	contig6_570044_571208	contig8_1982250_1984259	
10	contig6_2125851_2128117	contig8_1982250_1984262	
11	contig6_253170_255387	contig8_1992505_1994547	
12	contig6_2125851_2129551		
13	contig6_1530029_1532514		
14	contig6_1530556_1535883		
15	contig6_578256_580471		
16	contig6_1533503_1535883		
17	contig6_1592062_1593885		
18	contig6_388795_390935		
19	contig6_156852_158154		
20	contig6_569526_569884		
21	contig6_2206403_2209102		

Table S3 Primers designed for qRT-PCR

**Primers**	**Sequences**	**TM (^{o}C)**	**GC%**
*his*3-1-F	AGTCCACGGAACTCCTAATCA	55	47
*his*3-1-R	AGCGGCTAAGTTGGTGTCT	56	52
WcCYP52-F	ATCTTTGCCAATCCTAACGG	52	50
WcCYP52-R	TGGGTCCGTCTTGTTCTTAA	59	60
WcCYP64-1-F	ATCTTGGTGGAACTGCTGTG	55	50
WcCYP64-1-R	AGCCAGAGTACGACAGACAC	56	55
WcCYP_FUM15-F	ATCTACCGGGACCTCCTAAT	56	50
WcCYP_FUM15-R	CCTCGTGTCCATGGTATAAA	56	55
WcCYP64-2-F	TTGGAGAAGAATAGTGCGAT	55	50
WcCYP64-2-R	AAGCTACCGCAGTACGTGTG	56	50
