# Supplementary material for: An omics-based characterization of Wolfiporia cocos reveals three CYP450 members involved in the biosynthetic pathway of pachymic acid
Source: Commun Biol. 2024 May 30;7:666. doi: 10.1038/s42003-024-06323-1 (PMC11139888; doi:10.1038/s42003-024-06323-1)
Supplement: Supplementary file 2 — Description of Additional Supplementary Files [file 42003_2024_6323_MOESM2_ESM.pdf]

Description of Additional Supplementary Files

File name: Supplementary Data 1
Description: Chromatographic and mass spectrometric data of 11 triterpenoids isolated from *W.cocos*

File name: Supplementary Data 2
Description: CYP450 genes annotated in the genome of *W. cocos*

File name: Supplementary Data 3
Description: The source data behind the graphs in the paper.
